# Osteogenesis evaluation of duck’s feet-derived collagen/hydroxyapatite sponges immersed in dexamethasone

**DOI:** 10.1186/s40824-017-0088-4

**Published:** 2017-02-23

**Authors:** Yeon Ji Kook, Dae Hoon Lee, Jeong Eun Song, Nirmalya Tripathy, Yoo Shin Jeon, Ha Yan Jeon, Joaquim M. Oliveira, Rui L. Reis, Gilson Khang

**Affiliations:** 1 Department of BIN Convergence Technology, Department of Polymer Nano Science & Technology and Polymer Fusion Research Center, Chonbuk National University, 567, Beackje-daero, Deokjin, Jeonju, 561-756 Republic of Korea; 2 3B’s Research Group –Biomaterials, Biodegradables and Biomimetics, University of Minho, Headquarters of the European Institute of Excellence on Tissue Engineering and Regenerative Medicine, Guimaraes, Portugal

**Keywords:** Duck’s feet-derived collagen, Hydroxyapatite, Sponges, Dexamethasone, Osteogenesis, Bone regeneration

## Abstract

**Background:**

The aim of this study was to investigate the osteogenesis effects of DC and DC/HAp sponge immersed in without and with dexamethasone.

**Methods:**

The experimental groups in this study were DC and DC/HAp sponge immersed in without dexamethasone (Dex(−)DC and Dex(−)-DC/HAp group) and with dexamethasone (Dex(+)-DC and Dex(+)-DC/HAp group). We characterized DC and DC/HAp sponge using compressive strength, scanning electron microscopy (SEM). Also, osteogenic differentiation of BMSCs on sponge (Dex(−)DC, Dex(−)-DC/HAp, Dex(+)-DC and Dex(+)-DC/HAp group) was assessed by SEM, 3-[4,5-dimethylthiazol-2-yl]-2,5-diphenyltetrazoliumbromide (MTT) assay, alkaline phosphatase (ALP) activity assay and reverse transcription-PCR (RT-PCR).

**Results:**

In this study, we assessed osteogenic differentiation of BMSCs on Duck’s feet-derived collagen (DC)/HAp sponge immersed with dexamethasone Dex(+)-DC/HAp. These results showed that Dex(+)-DC/HAp group increased cell proliferation and osteogenic differentiation of BMSCs during 28 days.

**Conclusion:**

From these results, Dex(+)-DC/HAp can be envisioned as a potential biomaterial for bone regeneration applications.

## Background

An ideal scaffold for tissue engineering and regenerative medicine should have the following characteristics such as biocompatibility, good porosity, pore size, sufficient mechanical strength and good cell attachment [1]. Above all, the scaffold containing cells and bioactive substance components plays critical roles, which supports the transplanted cells and maintains bio-functions effectively [2–9]. Therefore, the methods of fabricating scaffolds with cells and bioactive molecules have been developed. The transplantation of artificial bone as the medical technology was significantly required to the patients who experienced the irreparable situation from accident or disease and so on [10]. Biomaterial interface in the scaffold plays important roles for cell adhesion, proliferation, migration, and differentiation [11]. Moreover, biomaterials for fabricating scaffolds can be adapted not only to be passively endured by the organism, but also to provide appropriate environment to help specific cell responses [12]. In this respect, biodegradable natural scaffolds are critical components in tissue engineering and choice of appropriate materials for scaffold can effectively enhance cell proliferation [7].

Collagen is generally used as a biomaterial for tissue engineering and has been known for diverse biomedical application in bone tissue engineering because of their excellent biocompatibility, low immunogenicity and biodegradability [13–20]. Moreover, collagen can be used in the various forms such as scaffold, film, hydrogel and porous sponge employing collagen solution [21–26]. Engineered collagen scaffold have been appropriate for osteogenesis and chondrogenesis of bone marrow-derived mesenchymal stem cells (BMSCs). Dexamethasone (Dex) is a synthetic glucocorticoid, which has been shown to promote cellular proliferation and well-known as an important regulator of mesenchymal progenitor cell commitment to lineages of osteoblast, chondrocyte and adipocyte. Moreover, it has been demonstrated that Dex controls the osteogenic differentiation of MSCs and mineralization in many studies [27–29].

In this study, we have evaluated osteogenic differentiation of BMSCs on duck’s feet-derived collagen (DC) sponges immerged in Dex (Dex(+)-DC). The sponges characteristic and its osteogenesis efficiency was studied using by SEM, 3-[4,5-dimethylthiazol-2-yl]-2,5-diphenyltetrazoliumbromide (MTT) assay, alkaline phosphatase (ALP) activity assay and reverse transcription-PCR (RT-PCR).

## Methods

Collagen was extracted from the duck’s feet purchased in local market of Korea. All the reagents and organic solvents were used HPLC grade purchased from Sigma-Aldrich (St Louis, MO, USA).

### Duck’s feet-derived collagen extraction

Duck’ feet derived collagen (DC) was prepared according to our previous reported study [30, 31]. First, the flippers were washed with distilled water and submerged in 0.5 M NaOH solution for 24 h to remove fat from tissue and washed with distilled water. All the flippers were again washed with methanol: chloroform (3: 1, V/V), acetone, ethanol and distilled water, respectively. For collagen extraction, flippers were crushed in a blender and added to 5% citric acid for 48 h. After the acid treatment, the supernatant was collected and centrifuged at 12,000 rpm for 15 min. Then the obtained precipitated collagen was treated with alcohol, lyophilized and pulverized to fine powder using Freezing Mill (6700 SPEX Inc., U.S.A.).

### Fabrication of DC and DC/HAp sponges

All the sponges were prepared by lyophilization. Briefly, DC powders were added in vial with solution consisting of 0.5 M acetic acid and stirred at room temperature for 24 h. 2% DC solution was poured onto 48-well plate (1 mL/well); refrigerated at 4 °C for 4 h; and frozen at −20 °C for 4 h and −75 °C for 24 h. 2% DC sponges were cross-linked with 0.25% glutaraldehyde solution for 24 h. The cross-linked DC sponges were washed distilled water and treated with 0.1 M glycine for 24 h to prevent the action of an aldehyde group. Prepared DC sponges were washed in distilled water and lyophilized for 48 h. DC/HAp sponges were prepared following the method: dried 2% DC sponges were immersed in 1.0X stimulated body fluid (SBF) solution for 24 h, washed in distilled water and lyophilized for 48 h.

### Characterizations of DC and DC/HAp sponges

The morphology of the sponges were evaluated by scanning electron microscopy (Bio-LV SEM, Hitachi, S-2250 N, Japan). The chemical characteristics of as-fabricated scaffolds were analyzed by FTIR Spectrometer (Spectrum GX, Perkin Elmer, USA) in the range of 4000 ~ 500 cm^−1^. Compressive strength of each scaffold was also measured using TMS-Pro instrument (Food Technology Corporation, Sterling, VA, USA). In order to calculate the strength and height of scaffolds, the samples were moved down at a target distance to specimen of 1.5 mm with a speed of 10 mm/s and force of 0.5 N.

### Cell culture

Bone marrow-derived mesenchymal stem cells (BMSCs) were isolated from New Zealand White rabbits (3 week old, female). Briefly, bone marrow was harvested from the tibia and femur condyle. BMSCs were isolated from bone marrow by ficoll density-gradient centrifugation. Then, the BMSCs were plated in culture medium, alpha-minimum essential medium (α-MEM) (Lonza, Walkersville, MD, USA) containing 20% fetal bovine serum, and 1% penicillin/streptomycin (Invitrogen, Carlsbad, CA, USA) at 37 °C and 5% CO_2_. The culture medium was replaced in every 3^rd^ day.

BMSCs were seeded onto each sponge at a concentration of 1 × 10^5^ cells per sponge. Before cell seeding, all sponges were sterilized in 70% ethyl alcohol for 30 min followed by rinsing it 3 times in phosphate buffer saline (PBS) solution for 5 min. To induce osteogenic differentiation of BMSC, osteogenic induction medium, culture medium with 1 mM Dex (Sigma-Aldrich), was applied after seeding of BMSC. In addition, we used culture medium without Dex for comparison in this study. Sponge called DC and DC/HAp sponge immersed culture medium (Dex(−)DC and Dex(−)-DC/HAp) and with Dex (Dex(+)-DC and Dex(+)-DC/HAp).

### In vitro cell adhesion and viability analysis

To examine cell adhesion and morphology of BMSCs on sponges, 10 μL of cell suspension was seeded in sponges having cell density of 5 × 10^4^ cells/sponges and cultured in culture medium without/with Dex for 7, 28 days in vitro. SEM analysis was carried out after treating samples with 2.5% SEM-grade glutaraldehyde (Sigma-Aldrich) for 24 h at room temperature followed by samples fixation with series of ethanol solutions (50, 60, 70, 80, 90, and 100%), and finally the images were observed by SEM (SN-SUPRA 40VP,Carl Zeiss, Germany). Adhered cells and scaffolds were overlaid with white gold of 200 μm in thickness using a plasma sputter (Emscope SC500 K, UK) under argon gas. The morphologies of cells on the sponges were examined by Bio-LV SEM (SN-3000 Hitachi, Japan).

The BMSCs proliferation on sponge in culture medium without/with Dex was evaluated by 3-[4,5-dimethylthiazol-2-yl]-2,5-diphenyltetrazoliumbromide (MTT) assay. At each time point (1, 7, 14, 21, and 28 days of cell culture), 100 μL MTT solution was added to the samples and incubated at 37 °C, 5% CO_2_ for 4 h. Then the medium was removed, sponges were washed with PBS followed by addition of 1 mL dimethyl sulfoxide (DMSO, Sigma-Aldrich) for 1 h at room temperature. The absorbance of samples was measured at 570 nm with a microplate reader (E-max, Molecular Device, USA). All the experiments were performed in triplicates.

### Cell osteogenic differentiation assay

The osteogenic differentiation of BMSCs on sponges was measured using ALP Assay Kit (Takara Bio Inc., Tokyo, Japan) manufacture’s protocol. BMSCs were seeded on sponge at concentration of 5 × 10^4^/sponge and cultured using culture medium without and with Dex for 1, 7, 14, 21 and 28 days. At each time point, the samples were rinsed with PBS, followed by the addition of 5% extraction solution to extract BMSCs from sponge and para-nitrophenyl phosphate (pNPP) solution. The extracted BMSCs were incubated at 37 °C and 5% CO_2_ for 1 h. The absorbance was read at 405 nm with a microplate reader (E-max, Molecular Device, USA). Also, osteogenic gene expression was evaluated by reverse transcription polymerase chain reaction (RT-PCR). After 1, 7, 14, 21 and 28 days of cell culture in sponges, total RNA was extracted using a Trizol reagent (Takara Bio Inc., Tokyo, Japan) and 0.2 mL chloroform. The supernatants precipitated with 0.5 mL of isopropanol (Sigma-Aldrich) and 5 μL of Polyacryl Carrier (Molecules Research Center, Inc., Cincinnati, OH, USA). The RNA samples were reverse transcribed into cDNA using Oligo (dT) primer (Invitrogen™), 5 ⨯ first strand buffer (Invitrogen™), dNTP (dGTP, dATP, dTTP, dCTP, Gibco), RNase inhibitor (Invitrogen™), SuperScript II (Intvirogen™), RNase H reverse transcriptase (Invitrogen™) and DNase/RNase free water (Gibco) by Authorized Thermal Cycler (TP 600, Takara Bio Inc, Japan). Each of primers; Glyceraldehyde 3-phosphate dehydrogenase (GAPDH), type-I collagen (Col-I), and osteocalcin (OCN) were extended using PCR Master kit (Roche, Germany) including 2 unit Taq DNA polymerase. We observed that all PCR products were separated via electrophoresis on 1.2% (w/v) agarose gel containing ethidium bromide (EtBr) and visualized under UV light (FluorChem HD2 Gel Imaging System, Alpha Innotech) at 300 nm. The primers used in this study was purchased from Genotec (Daejon, Korea).

### Statistical analysis

Data are presented as mean ± standard deviation (SD). Statistical analyses were performed based on Student’s *t*-test (Excel 2010, Microsoft) and the differences were considered significant where p* < 0.05, p** <0.005 and p*** <0.001.

## Result and discussion

### Structural and mechanical properties of DC, DC/HAp sponge

**Fig. 1:**
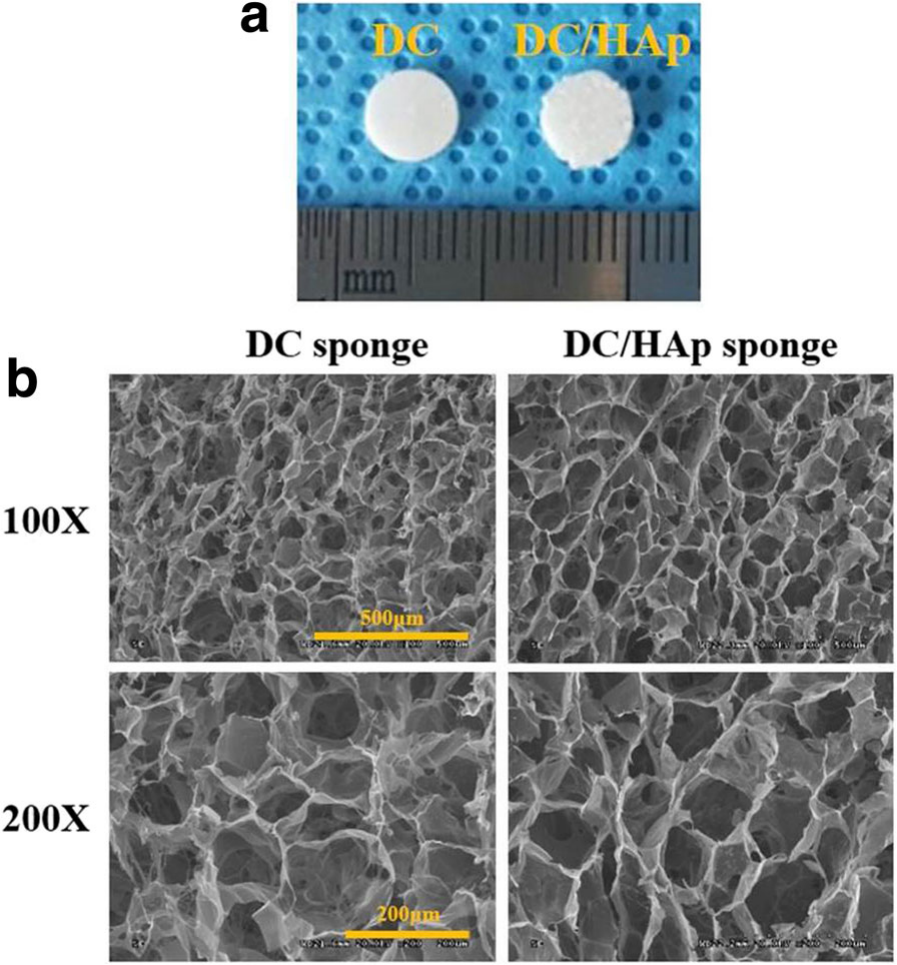
**a** Gross image of as-fabricated 2% DC, DC/HAp sponeges (**b**) Cross sectional SEM images of 2% DC sponges and DC/HAp sponges

Cross-sectional 3D structure and gross images of 2% DC and 2% DC/HAp sponges were observed by scanning electron microscopy in Fig. 1(a and b). Each sponge was fabricated with a 6 mm size and 1 mm thickness, as shown in Fig. 1a. The sponges were synthesized by freeze-drying method. As shown in the fig. 1b, the interconnected pores were successfully formed in both DC and DC/HAp sponges. The sponges display pore morphologies with concave and convex shapes. DC and DC/HAp sponges had similar pore sizes ranging from several micrometers and few hundred micrometers. These results suggest that DC, DC/HAp sponges could be suitable for cell growth and nutrient exchange.

**Fig. 2:**
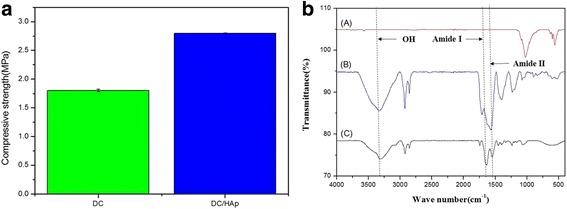
**a** Compressive strength and (**b**) FTIR of DC, DC/HAp sponges

Figure 2a shows the comprehensive strength analyzed for as-fabricated sponges. As compared to the comprehensive strength of DC sponge (1.8 MPa), the DC/Hap sponges showed about 1.5 times higher comprehensive strength i.e. 2.8 MPa. It is well-known that hydroxyapatite belongs to a family of compounds known as apatites that closely resemble mineral component of bone and thus can mimic natural bone [32]. Hence we assume that DC sponge coated HAp can be a suitable candidate as bone regeneration materials.

The FTIR spectrum of 2% DC displays several characteristic peaks at 1780 cm^−1^ and 600 cm^−1^ which can be ascribed to amide-I and amide-II, respectively. The FTIR spectrum of hydroxyapatite shows the peaks at 1114 cm^−1^ and 1002 cm^−1^ assigned to P-O phosphate groups stretching of three, and peaks at 968 cm^−1^ and 943 cm^−1^ were attributed to P-O bonds banding modes in the phosphate groups. Moreover, the FTIR spectra HAp show well-preserved characteristic peaks within the DC/HAp sponges, thus suggesting no significant structural changes of DC/HAp throughout the composite formation.

### In vitro assay (cell culture and proliferation)

**Fig. 3:**
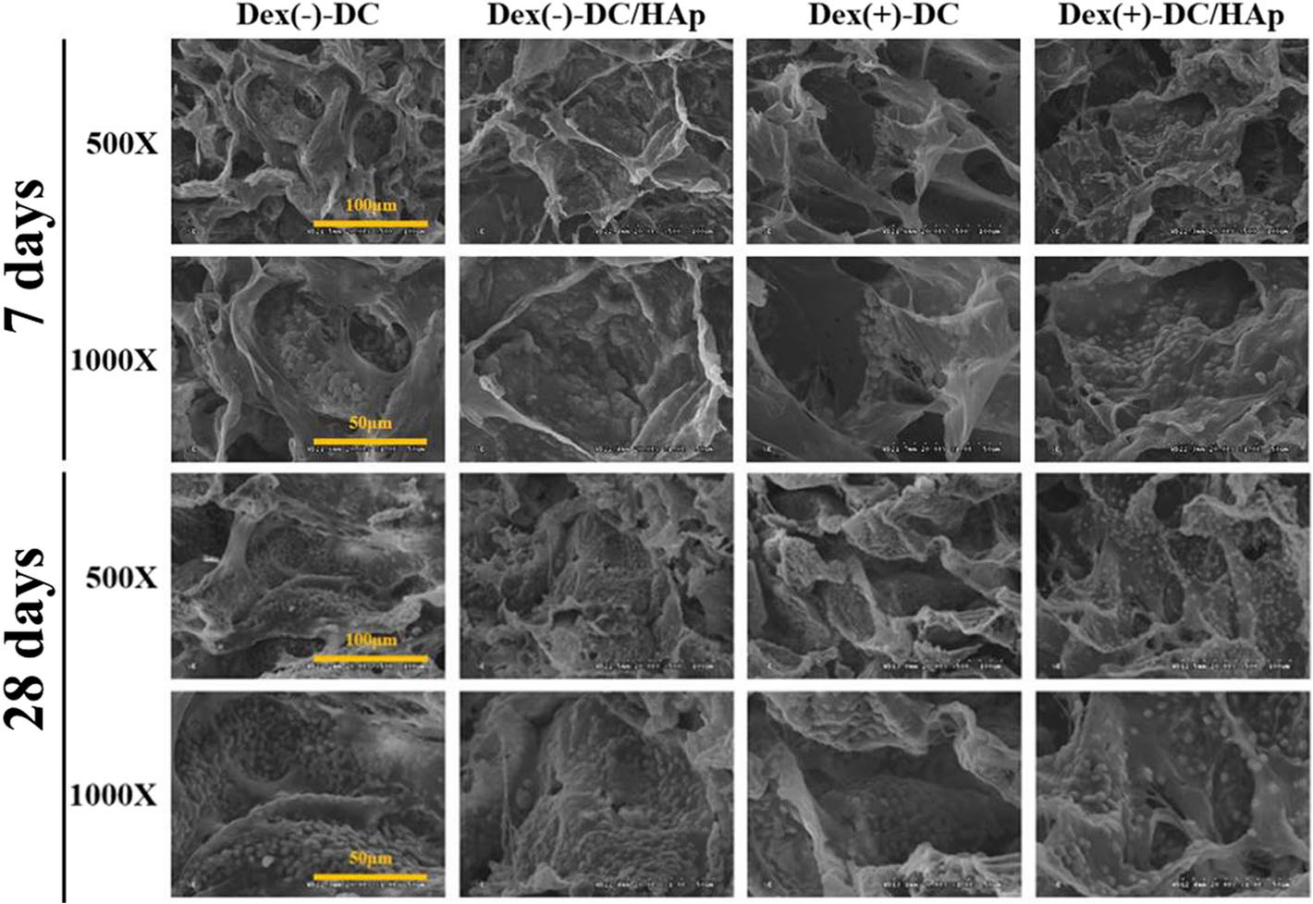
In vitro BMSCs behavioral study. SEM images of BMSCs on DC and DC/HAp sponges immersed dexamethasone during 7 and 28 days of culturing showing the cellular attachment and migration

Figure 3 shows the cell proliferation, attachment and migration test, BMSCs were generally cultured for 7 and 28 days onto the Dex(−)DC, Dex(−)-DC/HAp, Dex(+)-DC, Dex(+)-DC/HAp sponges. Overall, all the groups showed that well-grown cells, however proliferation of BMSC is not quite increased during culture period [33]. These findings indicated that collagen sponges and hydtoxyaptite coating can provide optimal condition for attachment and proliferation of BMSCs.

### MTT and ALP assay

**Fig. 4:**
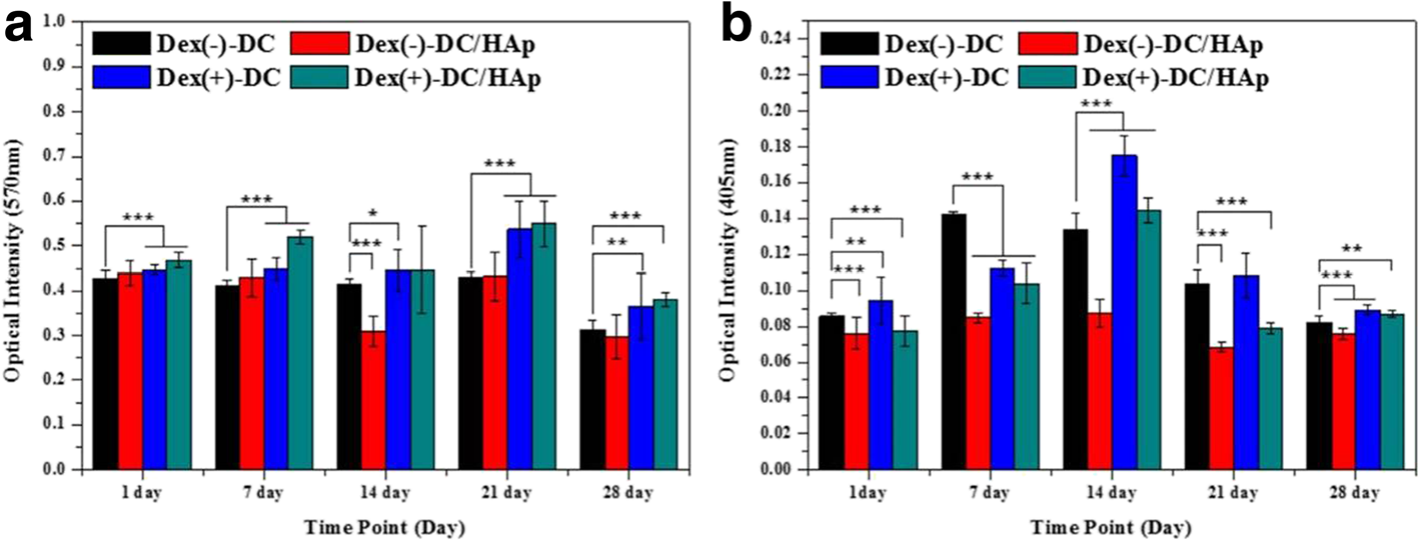
**a** Proliferation of BMSCs on DC sponges and DC/HAp immersed in dexamethasone after 1, 7, 14, 21, and 28 days studied by MTT assay. **b** Osteogenic differentiation of BMSCs on DC sponges and DC/HAp sponges immersed in Dex after 1, 7, 14, 21, and 28 days by ALP assay. (p* < 0.05, p** < 0.01, p*** < 0.001)

Figure 4 (a and b) shows the MTT and ALP assay measured in vitro over 28 day after seeding BMSCs on Dex(−)-DC, Dex(−)-DC/HAp, Dex(+)-DC, Dex(+)-DC/HAp sponges, respectively. The MTT results (Fig. 4a) showed an overall increase in cellular proliferation of BMSCs during entire culture period, although the increase is not significantly high. Furthermore, we observed a decreased optical density at 28 day due to lack of spaces in which cells are able to live continuously. Proliferation of BMSCs on the Dex(+) sponges was increased compared to the Dex(−) sponges. It can be seen that Dex(+) sponges can affect more cell culture medium. On the other hand, Compared to (+)Dex-DC and Dex(+)-DC/HAp sponges, proliferation of BMSCs was also increased Dex(+)/HAp sponges during culture period. Especially, Dex(+)-DC/HAp sponge tended to elevate proliferation of BMSCs than other sponges. These results indicate that HAp also can affect osteogenic differentiation of BMSCs. Moreover, we measure the ALP activity for each sponges and shown in Fig. 4b. An increased ALP activity was found for Dex(+) sponges compared to than Dex(−) sponges, presenting a consistency with the MTT assay. On the other hands, compared to DC sponge, the DC/HAp sponge showed decreased ALP activity. These results indicate that HAp coating can affect cell holding at the HAp coated sponges. ALP activity of BMSCs was elevated in Dex(+)-DC sponge more than other groups.

### Gene expression

**Fig. 5:**
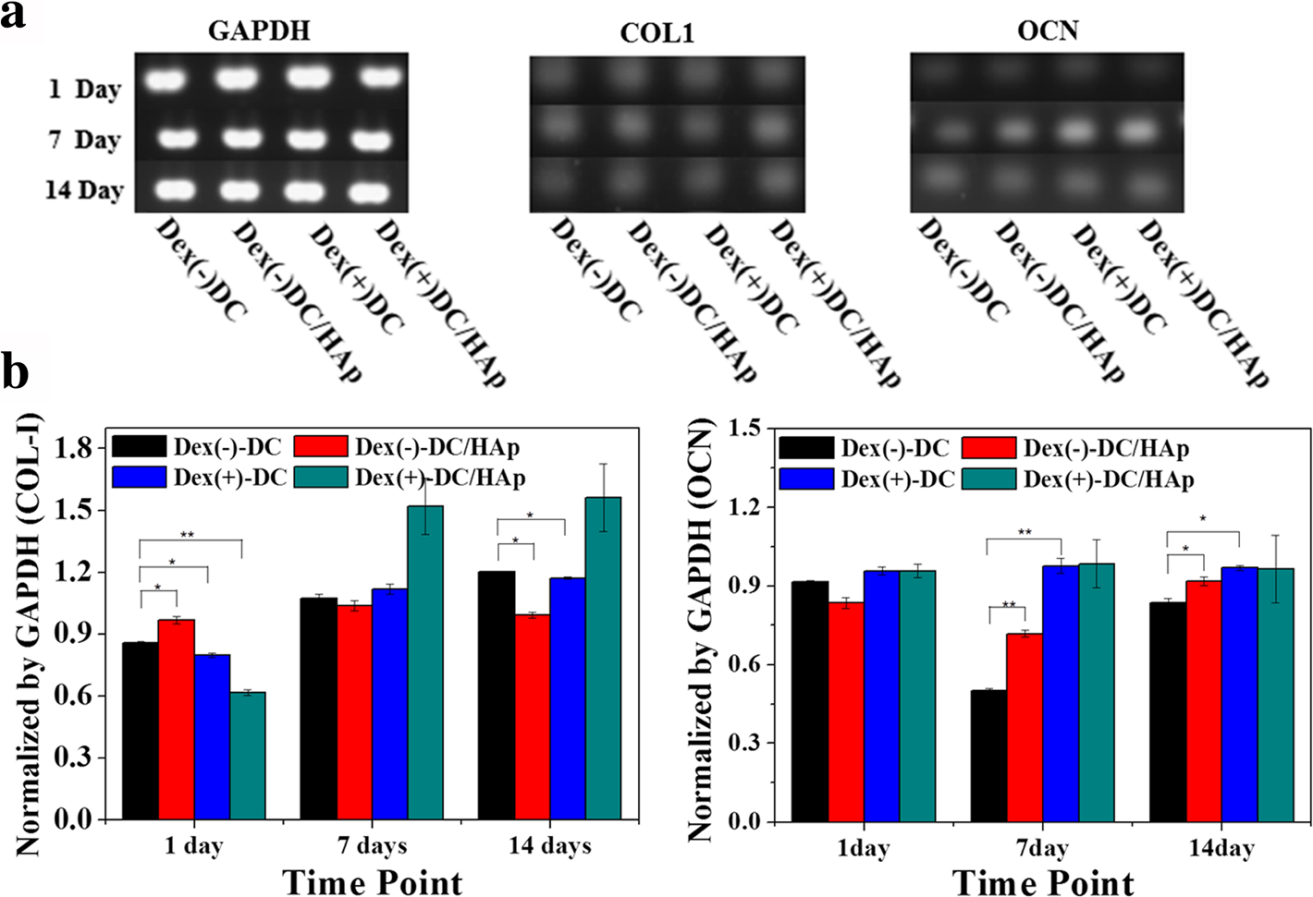
**a** mRNA expression of BMSCs. RT-PCR analysis of the BMSCs on sponges immersed dexamethasone after 14 days. RT-PCR results for GAPDH, Col I, OCN. **b** Quantitative analysis of Col I, OCN by normalization of GAPDH after 14 days

The effects of dexmethasone immersed sponges to raise osteogenic ability of BMSCs were investigated by gene expression of Col-I, OCN as bone-specific cytokines and these results were normalized by GAPDH as house-keeping gene, shown in Fig. 5. The gene expression of all cytokines related osteogenesis quantified after 14 days (Fig. 5b). When the results of gene expression were normalized by expression of GAPDH, gene expression of Col-I, OCN in all sponges was gradually increased. Above all, gene expression in Dex(+)-DC sponges was fairly elevated in comparison with other Dex(−)-DC sponges (Fig. 5b). The most elevated group was Dex(+)-DC/HAp. It is considered that Dexamethasone and HAp affected osteogenesis through collagen sponges and osteogenic differentiation of BMSCs.

## Conclusion

In conclusion, we have successfully fabricated duck’s feet collagen sponges and coated HAp sponges by immersing in HAp solution. BMSC was cultured on theses sponges in Dex(+), Dex(−) medium. The DC/HAp sponges displayed better compressive strength than only DC sponges. Furthermore, BMSC on Dex/DC/HAp sponge showed higher proliferation compared to other sponges. As well, we found enhanced osteogenic differentiation in Dex(+)-DC/HAp sponges with high ALP activity, col-I and osteocalcin expressions. Overall, the collagen, HAp, dexamethasone can be suggested as good bone tissue engineering materials. Especially Dex(+) medium showed high inclination towards bone formation in vitro. In conclusion, collagen, HAp, Dexamethasone had potentials as a good bone tissue engineering materials and can employed for devising improved versions of 3D culture base.

## Abbreviations

BMSCs: Bone marrow-derived mesenchymal stem cells
DC: Duck’ feet derived collagen
PBS: Phosphate buffer saline
RT-PCR: Reverse transcription polymerase chain reaction
SEM: Scanning electron microscope
α-MEM: Alpha-minimum essential medium
